# The Possible Role of Flavonoids in the Prevention of Diabetic Complications

**DOI:** 10.3390/nu8050310

**Published:** 2016-05-20

**Authors:** Roberto Testa, Anna Rita Bonfigli, Stefano Genovese, Valeria De Nigris, Antonio Ceriello

**Affiliations:** 1 Experimental Models in Clinical Pathology, INRCA-IRCCS National Institute, Ancona I-60127, Italy; r.testa@inrca.it; 2 Scientific Direction, INRCA-IRCCS National Institute, Ancona I-60127, Italy; a.bonfigli@inrca.it; 3 Department of Cardiovascular and Metabolic Diseases, IRCCS Multimedica, Sesto San Giovanni I-20099, Italy; stefano.genovese@multimedica.it; 4 Insititut d’Investigacions Biomèdiques August Pi i Sunyer (IDIBAPS), C/Rosselló, 149-153, 08036 Barcelona, Spain; vnigris@clinic.ub.es; 5 Centro de Investigación Biomédica en Red de Diabetes y Enfermedades Metabólicas Asociadas (CIBERDEM), Barcelona 08036, Spain

**Keywords:** type 2 diabetes mellitus, diabetic complications, flavonoids

## Abstract

Type 2 diabetes mellitus is a disease that affects many metabolic pathways. It is associated with insulin resistance, impaired insulin signaling, β-cell dysfunction, abnormal glucose levels, altered lipid metabolism, sub-clinical inflammation and increased oxidative stress. These and other unknown mechanisms lead to micro- and macro-complications, such as neuropathy, retinopathy, nephropathy and cardiovascular disease. Based on several *in vitro* animal models and some human studies, flavonoids appear to play a role in many of the metabolic processes involved in type 2 diabetes mellitus. In this review, we seek to highlight the most recent papers focusing on the relationship between flavonoids and main diabetic complications.

## 1. Introduction

**Figure 1:**
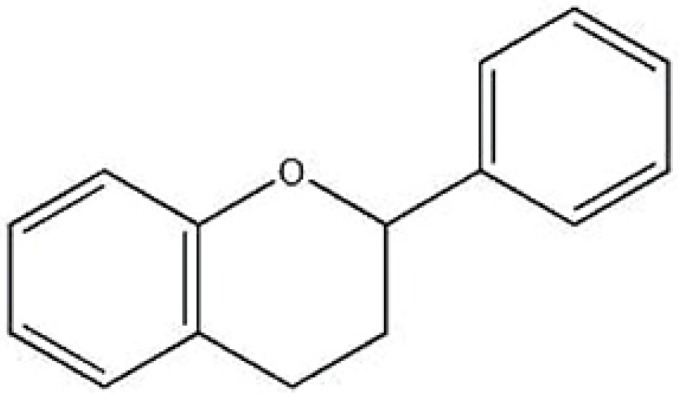
Basic chemical structure of a flavonoid.

Flavonoids are a large family of compounds that possess a common chemical structure (Figure 1) and are synthesized by plants. Flavonoids may be further divided into subclasses (Table 1) [1]. Recent studies provide growing evidence that polyphenols from plant foods, due to their biological properties, may be unique nutraceuticals and represent supplementary treatments for various aspects of type 2 diabetes mellitus (T2DM) [2] This article reviews the scientific evidence for the hypothesis that dietary flavonoids prevent or attenuate type 2 diabetic complications.

## 2. Flavonoids and Diabetes

Type 2 diabetes is a complex metabolic disorder. It is associated with insulin resistance (IR), impaired insulin signaling, β-cell dysfunction, abnormal glucose levels and altered lipid metabolism. It is also linked to sub-clinical inflammation and increased oxidative stress. These metabolic disorders lead to long-term pathogenic illnesses including micro- and macro-vascular complications, neuropathy, retinopathy, nephropathy, and, consequently, decreased quality of life and increased rate of mortality [3,4,5]. Linked to their biological properties, polyphenols may be useful nutraceuticals and supplementary treatments for various aspects of diabetes mellitus. Based on several *in vitro* animal models and some human studies, polyphenols may play a role in many metabolic processes. They can modulate carbohydrate and lipid metabolism, attenuate hyperglycemia, dyslipidemia and insulin resistance, improve adipose tissue metabolism, and alleviate oxidative stress and stress-sensitive signaling pathways and inflammatory processes [2,6,7].

In this review, we seek to highlight the most recent papers that have focused on the relationship between flavonoids and main diabetic complications.

## 3. Diabetic Neuropathy

Diabetic peripheral neuropathy (DPN) is a frequent and severe complication of diabetes mellitus, and a cause of considerable morbidity [8]. Chronic sensorimotor DPN is the most common form. In this syndrome, longer nerve fibers are affected to a greater degree than shorter ones because the nerve conduction velocity (NCV) is slowed in proportion to nerve length [9]. In clinical examination, the lower limbs usually reveal abnormal sensations of vibration, pressure, pain, and temperature perception [7]. Research on the morphological changes in DPN has focused mainly on the peripheral nerves, such as axonal degeneration, demyelination, Schwann cell pathology, *etc.* [8]. Involvement of the spinal cord in patients with early DPN has also been demonstrated [10]. Therefore, determining the extent of the involvement of the nervous system is important in order to achieve a better understanding of the pathogenesis of DPN. Unfortunately, the inner mechanisms underlying DPN remain unknown. It is believed to be a multifactorial pathology involving hyperglycemia, dyslipidemia, oxidative stress, mitochondrial dysfunction, and loss of calcium homeostasis [11,12,13,14]. In this section, we discuss whether early treatment of peripheral neuropathy with phytochemical approaches may be important in preventing the progression of diabetic complications. **Flavanols**. An interesting paper found alleviation of DPN in rats with T2DM by early intervention with grape seed proanthocyanidins. They also preserve normal morphology of nervous tissues (L4 to L5 spinal cord segments, L5 DRG, and sciatic nerves), alleviating hyperglycemia, and reversing Ca2+ overload by increasing Ca2+ -ATPase activity in sciatic nerves [15]. Another study on proanthocyanidins was performed in Sprague–Dawley T2DM rats with low-dose streptozotocin and a high-carbohydrate/high-fat diet, and in rat Schwann cells cultured in serum from type 2 diabetic rats. The administration of grape seed proanthocyianidins significantly decreased low-density lipoprotein levels and increased nerve conduction velocity in diabetic rats [16]. Another flavanol, epigallocatechin-gallate (EGCG), is an antioxidant with an efficacy similar to that of alpha-lipoic acid in protecting cellular DNA from reactive oxygen species (ROS) but with a much higher potency in reducing lipid peroxidation [17,18]. Raposo *et al.* demonstrated that diabetes induces oxidative stress in nociceptive spinal cord neurons located in laminae I–III and that an early antioxidant treatment with EGCG can prevent certain long-term effects of the disease, namely oxidative stress damage and neuronal hyperactivity at the spinal cord, and ameliorate behavioral signs of DNP [19]. **Flavonols.** Quercetin is a bioflavonoid found in red wine and numerous fruits, vegetables, and nuts [20,21]. A recent study investigated the effect of quercetin supplementation on the myenteric neurons and glia in the cecum of diabetic rats, demonstrating a neuroprotective effect [22]. Quercetin has also been demonstrated to protect rat cultured dorsal root ganglion neurons against high glucose-induced injury *in vitro* through Nrf-2/HO-1 activation and nuclear factor K beta (NF-κB) inhibition, which may prove beneficial for the treatment of diabetic neuropathy [23]. A randomized, placebo-controlled, double-blind trial on men and women with type 1 or 2 diabetes and diabetic neuropathy who applied QR-333, a topical compound that contains quercetin, or placebo three times daily for 4 weeks, to each foot where symptoms were experienced, demonstrated that QR-333 significantly relieved symptoms of diabetic neuropathy, improved quality of life, and was safe and well tolerated [24]. QR-333 reduced the severity of numbness, jolting pain, and irritation from baseline values [24]. **Isoflavones.** These were studied in a randomized double-blind, placebo controlled study comparing soy germ pasta with conventional pasta for effects on gastric emptying. The major findings of this pilot study on T2DM patients with gastroparesis were that the inclusion of an isoflavone-enriched soy germ pasta in an ADA diet led to a significant acceleration in the rate of gastric emptying [25]. Puerarin, a major isoflavonoid derived from the Chinese medical herb radix puerariae, has been reported to be useful in the treatment of many diseases [26,27]. The clinical efficacy and safety of puerarin injection was evaluated in a clinical review [28]. Randomized controlled trials investigating the efficacy of puerarin injection on DPN were searched, and twenty-two studies involving 1664 participants were included. Puerarin injection was effective for the treatment of DPN. Puerarin can improve the total effective rate, correct nerve conduction velocity that has been decreased by diabetes, and improve the hemorheology index. Puerarin was also found to be relatively safe clinically. **Flavones.** Naringenin (NA), a flavones flavonoid, is a biologically active molecule found in citrus fruits such as grapefruits and oranges [29]. NA neutralises oxidative stress and nerve growth factor discrepancy in experimental diabetic neuropathy [30]. Hassanein *et al.* demonstrated that long-term NA administration was able to dose-dependently elicit significant anti-hyperalgesic, anti-allodynic, and hypoglycemic effects in a rat model of diabetic neuropathy [31]. Baicalein (5,6,7-trihydroxyflavone), a flavonoid originally isolated from the roots of *Scutellaria baicalensis*, has been employed for many centuries in traditional Chinese herbal medicine as an antibacterial and antiviral remedy [32]. Baicalein alleviates diabetic peripheral neuropathy through inhibition of oxidative–nitrosative stress and p38 MAPK activation. In particular, the flavonoid baicalein alleviates motor and sensory nerve conduction velocity deficits, thermal hypoalgesia, and tactile allodynia characteristic for DPN, without slowing down diabetes-associated loss of intraepidermal nerve fibers and promoting their regeneration [33]. Regarding DPN, also diabetic erectile dysfunction associated with penile dorsal nerve bundle neuropathy in the corpus cavernosum was evaluated in rats treated with Icariside II, a flavonols isolated from herba epimedii. Diabetic animals exhibited a decreased density of the dorsal nerve bundle in the penis. The neurofilament of the dorsal nerve bundle was fragmented in diabetic rats. There was a decreased expression of nNOS and NGF in the diabetic group; however, the ICA II-treated group had a higher density of the dorsal nerve bundle and a higher expression of NGF and nNOS in the penis [34]. **Flavanones.** Hesperidin is a flavanone glycoside found in citrus fruits. In combination with insulin, hesperidin has been found to not only improve the diabetic condition but also to reverse neuropathic pain, via control over hyperglycemia and hyperlipidemia, to down-regulate the generation of free radical release of pro-inflammatory cytokines as well as increases in membrane-bound enzyme [35]. Diosmin, a natural flavonoid glycoside, is readily obtained by dehydrogenation of hesperidin. It is abundant in the pericarp of various citrus fruits. A study was undertaken to evaluate the effect of diosmin on diabetic neuropathy in type 2 diabetic rats. Rats were fed with a high-fat diet throughout the experiment schedule, and administration of low-dose streptozotocin induced significant elevation of blood glucose levels and insulin resistance. Treatment with diosmin at doses of 50 and 100 mg/kg significantly restored the reduced body weight, elevated blood sugar and lipid profiles. Furthermore, the dose-dependent improvement was also observed in thermal hyperalgesia, cold allodynia and walking function in diabetic rats treated with diosmin. Elevated levels of malondialdehyde and nitric oxide, and decreased glutathione levels and superoxide dismutase activity, were restored significantly in diabetic rats after 4 weeks of diosmin treatment. In conclusion, diosmin has shown beneficial effects in preventing the progression of early diabetic neuropathy in rats [36]. **Curcumin** is a naturally occurring compound that is extracted from the roots of Curcuma longa. Curcumin exhibits multiple pharmacological properties, including antioxidant, anti-carcinogenic, and anti-inflammatory in different models of rodents [37]. Curcumin promotes nerve regeneration and functional recovery after sciatic nerve crush injury in diabetic rats [38]. In conclusion, we have highlighted that animal and cellular studies, and some *in vivo* studies suggest that flavonoids have the potential to be used as therapeutic agents in diabetic neuropathy.

## 4. Diabetic Retinopathy

Diabetic retinopathy (DR) is a potentially devastating disease and is the most common cause of blindness among working-age people in the world [39]. It is a multifactorial progressive disease affecting neuro and glial cells and vascular elements of the retina [40,41,42]. In this section, we briefly show different molecules that have been tested to improve retinal damage. Cocoa is rich in flavonoids and has gained attention due to evidence that they lower blood pressure (BP) and improve endothelial function [43,44,45]. Duarte *et al.* demonstrated that cocoa enriched with polyphenol improves the retinal SIRT-1 pathway, thereby protecting the retina from diabetic milieu insult in rats [46]. Another study on flavonoids evaluated data from 381 participants with diabetes from the National Health and Nutrition Examination Survey (NHANES), 2003–2006. Blood samples were taken to measure C-reactive protein (CRP), HbA1C, and fasting glucose and insulin. Diabetic retinopathy was assessed from a retinal imaging exam. A high-flavonoid fruit and vegetable consumption (HFVC) index variable was created from a food frequency questionnaire. The results showed that greater HFVC was associated with lower levels of CRP, HbA1C and glucose, with greater HFVC reducing the odds of having diabetic retinopathy by 30% [47]. **Flavanones.** Eriodictyol is an antioxidative flavonoid extracted from Eriodictyon californicum, commonly called California Yerba Santa, a plant native to North America. Eriodictyol attenuates the degree of retinal inflammation and plasma lipid peroxidation, preserving the blood–retinal barrier in early diabetic rats [48]. Hesperidin is a compound with a flavonone glycoside chemical structure, which is abundant in citrus fruits. In streptozotocin-induced diabetic rats, hesperidin treatment significantly suppressed blood-retina breakdown and increased retina thickness, reduced blood glucose, aldose reductase activity and retinal TNF-α, ICAM-1, VEGF, IL-1β and AGEs levels [49]. **Flavanols.** Epicatechin is a monomeric flavonoid. Epicatechin is found in high concentrations in many fruits and vegetables, in particular, in cocoa and green tea [50,51]. Epicatechin was able to break down pre-formed glycated human serum albumin *in vitro*, as well as to reduce AGE accumulation in retinas *in vivo* in a dose-dependent manner [52]. In exogenously AGE-injected rats, treatment with epicatechin was evidenced by an improved retinal vascular apoptosis [52]. Epigalloccatechin-3-gallate (EGCG) is the main polyphenol component of green tea (leaves of *Camellia sinensis*). It inhibits ocular neovascularization and vascular permeability in human retinal pigment epithelial and human retinal microvascular endothelial cells via suppression of MMP-9 and VEGF activation [53]. **Flavonols.** Myricetin (3,5,7,30,40,50-hexahydroxyflavone, Cannabiscetin) is a primitive flavonoid from the Chrysobalanaceae family and found in most berries, vegetables, and various medicinal herbs [54]. Myricetin inhibits advanced glycation end product (AGE)-induced migration of retinal pericytes through phosphorylation of ERK1/2, FAK-1, and paxillin *in vitro* and *in vivo* [55]. Among flavonoids, the most common native flavonoid is rutin (3,30,40,5,7-pentahydroxyflavone-3-rhamnoglucoside), the main glycoside (a 3-orhamnoglucoside) form of quercetin, which is abundantly present in foods including onions, apples, tea, and red wine [56]. In Wistar rats treated with streptozotocin, rutin possesses antidiabetic activity, as blood glucose levels decreased and insulin levels increased. In the diabetic retina, rutin supplementation enhanced the reduced levels of brain-derived neurotrophic factor, nerve growth factor, and glutathione, and reduced the level of thiobarbituric acid-reactive substances [57]. Icariin (ICA) is thought to be the principal active moiety of Epimedii herba. A study investigated the effect of ICA supplementation on diabetic retinopathy in a streptozotocin-induced diabetic rat model system [58]. Numerous pathological changes (deceased expression of RECA, VEGF, Thy-1, and Brn3a, as well as decreased Collagen IV and Müller cell content) were noted in the retinal vessels of diabetic rats; these changes were attenuated in diabetic animals that received ICA [58]. **Isoflavones.** Puerarin can decrease retina pigment epithelial cell apoptosis in diabetic rats by reducing peroxynitrite level and iNOS expression, thus serving as a potential therapeutic agent in the control of diabetic retinopathy [59]. Another study evaluated the protective effect of puerarin against IL-1β-induced cell dysfunction in TR-iBRB2 cells, a retinal capillary endothelial cell line. Data showed that puerarin attenuated IL-1β-mediated leukostasis and cell apoptosis in TR-iBRB2 cells [60]. Isoflavones, which belong to the subfamily of isoflavonoids, comprise of a class of organic compounds, often naturally occurring [61]. Pentamethoxy-5,30-dihydroxyflavone has shown significant inhibition of rat retina aldose reductase in a non-competitive manner [62]. **Anthocyanins** are important natural bioactive pigments responsible for the red-blue color of fruits, leaves, seeds, stems and flowers in a variety of plant species [63]. Anthocyanins are an important natural antioxidant pigment known to be health-promoting in degenerative diseases; in particular, they seem to be an alternative therapy to diabetic retinopathy [63]. **Flavones.** Scutellarin is a flavone, a type of phenolic chemical compound. It can be found in *Scutellaria barbata* and *S. lateriflora*. Scutellarin inhibits high glucose-induced and hypoxia-mimetic agent-induced angiogenic effects in human retinal endothelial cells through reactive oxygen species/hypoxia-inducible factor-1α/vascular endothelial growth factor pathway [64]. **Silybin**, a bioactive polyphenolic flavonoid in the seeds of milk thistle (*Silybum marianum*), has been used as a traditional drug for over 2000 years to treat a range of liver disorders [65,66]. A recent study showed that silybin treatment significantly prevented the development of obliterated retinal capillaries in diabetic rats. In addition, leukostasis and level of the retinal ICAM-1 were found to decrease considerably in silybin-treated diabetic rats [67]. In conclusion, evidence from animal and cellular studies as well as cross-sectional data from NHANES 2003-2006 provide evidence for a beneficial effect of flavonoids in diabeticretinopathy. The molecular and cellular mechanisms of these molecules are not fully elucidated, requiring further studies.

## 5. Diabetic Nephropathy

Diabetic nephropathy can result in a progressive decline in glomerular filtration rate characterized by glomerular hyperfiltration, glomerular and tubular epithelial hypertrophy, increased urinary albumin excretion, increased basement membrane thickness and mesangial expansion with the accumulation of extracellular matrix (ECM) proteins [68]. The progression of renal injury often leads to end-stage renal disease, affecting 20%–40% of diabetic patients [69]. In this section, we examine *in vitro* and *in vivo* studies in which a phytochemical approach was used to prevent or mitigate diabetic nephropaty. Garcinia kola seeds (Family: Guttiferae) are consumed in western and central Africa and contain high biflavonoid levels, which have several pharmacokinetic advantages over simple monomeric flavonoids, as they survive first-pass metabolism [70]. **Kolaviron** (KV) is a mixture of flavonoids extracted from the seeds of Garcinia kola, which has numerous therapeutic effects. Kolaviron treatment on diabetic rats restored the activities of antioxidant enzymes, reduced lipid peroxidation and increased ORAC and GSH concentration in renal tissues. Kolaviron treatment on diabetic rats also suppressed renal IL-1 [71]. **Flavanols.** Grape seed proanthocyanidin extract (GSPE) is extracted from grape seeds and skins. Diabetic rats treated with GSPE demonstrated decreased fasting blood glucose, serum insulin, HbA1c and systolic blood pressure. GSPE significantly improved renal function parameters, reduced the expression of tissue inhibitor of metalloproteinase-1 and also increased the activity of matrix metalloproteinase-9 [72]. Oligonol is a phenolic product derived from lychee fruit extract containing catechin-type monomers and oligomers of proanthocyanidins, produced by a manufacturing process, which converts polyphenol polymers into oligomers. Oligonol was able to improve diabetic indices, prevent the development of diabetic renal disease, and preserve renal cells and renal morphological structure via the attenuation of reduced nicotinamide adenine dinucleotide phosphate oxidase-induced oxidative stress, inhibition of advanced glycation end-product generation, and prevention of apoptosis-induced cell death in db/db mice [73]. **Chalconoids.** Chalcone, a group of naturally occurring compounds that belong to the flavonoid family, is present in a variety of plant species, including fruits, vegetables, spices, and tea. A novel compound (E)-2,3-dimethoxy-4′-methoxychalcone (L6H21,) was found to exhibit strong inhibitory activity against inflammatory cytokine releases induced by lipopolysaccharides [74]. A study conducted by Fang *et al.* investigated whether L6H21 could ameliorate high glucose-mediated inflammation in NRK-52E cells and attenuate the inflammation-mediated renal injury. L6H21 showed a great inhibitory effect on the expression of pro-inflammatory cytokines, cell adhesion molecules, chemokines, and macrophage adhesion, via down-regulation of NF-κB/MAPKs activity in high-glucose-stimulated renal NRK-52E cells. Moreover, *in vivo* oral administration with L6H21 showed decreased expression of proinflammatory cytokines -cell adhesion molecules which subsequently contributed to the inhibition on renal macrophage infiltration-, reduction of serum creatinine and BUN levels, and improvement in the fibrosis and pathological changes in renal tissues of diabetic mice [75]. **Flavonols.** A study demonstrated that chronic oral treatment with low-dose quercetin exerts antidiabetic effects and attenuates the development of nephropathy in STZ-induced DN mice. These results are supported by a decrease in plasma glucose, creatinine, triglycerides, proteinuria and diminution of mesangial matrix expansion, accompanied by a reduction in •O_2_^−^ production and apoptosis in kidney cells [76]. **Isoflavones.** Genistein, a phyto-esterogen with isoflavone structure, is found in a wide variety of plant-derived foods, in particular, soybeans. Regarding genistein, a study highlighted that fructose-fed rats displayed significant elevation in BP and heart rate. Significant increases in plasma angiotensin-converting enzyme (ACE) activity, alterations in renal lipid profile, nitrite and kallikrein activity, enhanced expression of membrane-associated PKC-bII, and decreased expression of eNOS were observed in them. Histology and electron microscopic studies showed structural changes in the kidney. Genistein administration lowered BP, restored ACE, PKC-bII and eNOS expression and preserved renal ultrastructural integrity [77]. One study focused on a micronized purified flavonoid fraction (MPFF), which possesses antioxidant properties [78,79]. MPFF contains diosmin (90%) and other related flavonoids-hesperidin, diosmetin, linarin and isorhoifolin [80]—registered as **Daflon**^R^ 500 mg (Servier). Daflon^R^ inhibits AGE formation, reduces urinary albumin clearance and corrects hypoalbuminemia in normotensive and hypertensive diabetic rats [81]. In conclusion, only animal and cellular studies provide evidence that flavonoids may be used as therapeutic agents in diabetic nefropathy. *In vivo* studies are necessary to confirm their role in this diabetic complication.

## 6. Diabetic Cardiovascular Disease

Diabetes mellitus is widely known to increase the morbidity and mortality of cardiovascular disease [82,83]. Compared with non-diabetic patients, diabetic patients are twice as likely to die after myocardial infarction (MI) [84]. Diabetic patients also have a higher incidence of heart failure (HF) after MI, having undergone traditional treatment and primary angioplasty [85]. In this section, we seek to highlight the most recent papers focusing on the relationship between flavonoids and diabetic cardiovascular complications. **Flavanones.** Hesperidin produces cardioprotective effects via the PPAR-c pathway in an ischemic heart disease model in diabetic rats. In particular, hesperidin pretreatment significantly improved mean arterial pressure, reduced left ventricular end-diastolic pressure, and improved both inotropic and lusitropic function of the heart as compared to controls [86]. **Flavanols.** As cited before, catechins are polyphenolic compounds obtained mainly from green tea leaves derived from the plant *Camellia sinensis* [87]. The key finding in the study of Bhardwaj *et al*. is that the protective effect of catechin in preventing diabetes mellitus-induced experimental vascular endothelial dysfunction in STZ-diabetic rats might be mediated through the activation of the PI3K and eNOS signaling system [88]. Anthocyanins, as a group of flavonoids, are most abundant in various colorful fruits, vegetables, red wine, and grains [89]. Anthocyanin cyanidin-3-O-β-glucoside increases serum adiponectin concentrations and improves endothelial function in diabetic mice [90]. Supplementation of cyanidin-3-o-b-glucoside also promotes endothelial repair and prevents enhanced atherogenesis in diabetic apolipoprotein E-deficient mice [91]. Dimeric procyanidin B2 (GSPB2) is one of the main components of grape seed procyanidin extracts. In one study, GSPB2 significantly prevented obesity in db/db mice. GSPB2 also prevented the elevation in the levels of serum AGEs observed in db/db mice, suggesting an anti-non-enzymatic glycation effect of GSPB2. Moreover, the elevated levels of triglycerides and total cholesterol were significantly reduced by GSPB2. Myocardial hypertrophy and cardiac matrix remodeling were suppressed by GSPB2 in the cardiac sections of db/db mice [92]. **Isoflavones**. Puerarin performs a series of beneficial activities on cardiovascular diseases such as myocardial infarction [93] and hypertension [94]. Puerarin improves cardiac function through regulation of energy metabolism in streptozotocin-nicotinamide-induced diabetic mice after myocardial infarction. In particular, puerarin was shown to significantly increase survival rate, improve cardiac function and increase expression and translocation of GLUT4, while it attenuated expression and translocation of CD36 [95]. **Astilbin**, an active flavonoid compound, is isolated from the rhizome of *Smilax china* L. (Smilaceae), which is widely used in traditional Chinese medical treatment [96]. An interesting study evaluated the anti-myocardial ischemia and reperfusion (I/R) injury effect of Astilbin on diabetic rats *in vivo* and elucidated the potential mechanism *in vitro*. Astilbin significantly attenuated hypoxia-induced cell injury in a concentration-dependent manner. Treatment of H9c2 cells with Astilbin blocked NF-κB phosphorylation by blocking high-mobility group box protein 1 (HMGB1) expression. Treatment of diabetic rats with Astilbin by intravenous injection protected the rats from myocardial I/R injury as indicated by decreasing infarct volume, improving hemodynamics and reducing myocardial damage, and also lowered serum levels of pro-inflammatory factors, reduced HMGB1 and phosphorylated NF-jB expression in ischemic myocardial tissue [97]. **Flavones**. A recent study investigated the possible protective effect of baicalein on both insulin deficiency (ID) and IR. ID and IR were induced by STZ or fructose in male Wistar rats. Both models resulted in elevated BP, increased vasoconstriction and impaired relaxation KCl, elevated TNF-α and AGEs, NF-κB activation, marked infiltration of leukocytes in the adventitia, pyknosis of endothelial cells and marked collagen deposition. Baicalein ameliorated elevations in BP in models, prevented an exaggerated vasoconstriction IR model and improved relaxation in an ID model. Baicalein reduced AGEs and TNF-α levels, decreased NF-κB activation and inhibited histopathological changes in both models [98]. Liquiritin is a flavone compound derived from *Glycyrrhizae radix*. Liquiritin attenuates advanced glycation end-product-induced endothelial dysfunction via the RAGE/NF-ĸB pathway in human umbilical vein endothelial cells [99]. **Flavonols.** Quercetin was shown to protect against diabetes-induced exaggerated vasoconstriction and reduce high blood pressure. In addition, quercetin inhibited diabetes-associated adventitial leukocyte infiltration, endothelial pyknosis and increased collagen deposition [100]. Taxifolin, also known as dihydroquercetin, is a flavonoid commonly found in *Pseudotsuga taxifolia*, Dahurian larch, and syn *Larix dahurica* Turoz (Pinaceae) [101]. In mice models of diabetes, taxifolin effectively ameliorated diastolic dysfunction and showed protective effects against H9c2 cells apoptosis induced by high glucose incubation [102]. **Silymarin** is a flavonoid mixture extracted from *Silybum marium*. Silymarin proved to block alloxan-induced decreases in the activity and changes in the expression levels of renal antioxidant enzymes, such as superoxide dismutase, glutathione peroxidase and catalase [103]. Tuorkey *et al*. investigated the protective effect of silymarin against apoptotic death of cardiomyocytes associated with diabetes in rats. As a result of treatment with silymarin, a marked reduction of immunoreaction for caspase-3 expression was observed in diabetic rats similar to that in the control group. The levels of glucose, creatinine, AST, ALT, cholesterol, and triglycerides declined in the treated rats. The declined levels of insulin were enhanced again after treatment of diabetic rats with silymarin, reflecting a restoration of pancreatic β-cell activity [104]. In conclusion, different therapeutical approaches have been suggested, particularly in *in vitro* studies, where flavonoids seem to have an effect on improving cardiovascular disease in diabetes mellitus.

## 7. Conclusions

We can therefore affirm that animal and cellular studies, and some *in vivo* studies, provide mounting evidence that a large body of flavonoids may have beneficial actions to fight diabetic complications. The molecular and cellular mechanisms, although not fully elucidated, involve a large quantity of biochemical pathways and are under active investigation. Further studies are needed to clarify the mechanisms underlying the action of flavonoids. These studies provide a strong rationale for well-powered, randomized placebo controlled intervention trials to be performed in patients with diabetic complications. Randomized controlled studies should also be performed in combination with current anti-diabetic drugs, as the action of flavonoids may be most effective as an adjunctive rather than a primary therapy. In our opinion, trials are needed for each of the cited diabetic complications, as they could provide a stronger foundation for future therapeutical approaches.

## Figures and Tables

**Table 1:** Major flavonoids cited in this review.

Flavonoid Subclass	Dietary Flavonoids	Some Common Food Sources
Anthocyanidins	Cyanidin	Red, blue, and purple berries; red and purple grapes; red wine
Flavanols	Monomers (Catechins): Catechin, Epicatechin, Epigallocatechin gallate Dimers and Polymers: Proanthocyanidins	Catechins: Teas (particularly green and white), chocolate, grapes, berries, apples Theaflavins, Thearubigins: Teas (particularly black and oolong) Proanthocyanidins: Chocolate, apples, berries, red grapes, red wine
Flavanones	Naringenin, Eriodictyol	Citrus fruit and juices, e.g., oranges, grapefruit, lemons
Flavonols	Quercetin, Myricetin	Widely distributed: yellow onions, scallions, kale, broccoli, apples, berries, teas
Isoflavones	Genistein	Soybeans, soy foods, legumes
